# RAGE-dependent potentiation of TRPV1 currents in sensory neurons exposed to high glucose

**DOI:** 10.1371/journal.pone.0193312

**Published:** 2018-02-23

**Authors:** Doris Lam, Zeinab Momeni, Michael Theaker, Santosh Jagadeeshan, Yasuhiko Yamamoto, Juan P. Ianowski, Verónica A. Campanucci

**Affiliations:** 1 Department of Physiology, College of Medicine, University of Saskatchewan, Saskatoon, Saskatchewan, Canada; 2 Department of Biochemistry and Molecular Vascular Biology, Kanazawa University Graduate School of Medical Science, Kanazawa, Japan; Hirosaki Daigaku, JAPAN

## Abstract

Diabetes mellitus is associated with sensory abnormalities, including exacerbated responses to painful (hyperalgesia) or non-painful (allodynia) stimuli. These abnormalities are symptoms of diabetic peripheral neuropathy (DPN), which is the most common complication that affects approximately 50% of diabetic patients. Yet, the underlying mechanisms linking hyperglycemia and symptoms of DPN remain poorly understood. The transient receptor potential vanilloid 1 (TRPV1) channel plays a central role in such sensory abnormalities and shows elevated expression levels in animal models of diabetes. Here, we investigated the function of TRPV1 channels in sensory neurons cultured from the dorsal root ganglion (DRG) of neonatal mice, under control (5mM) and high glucose (25mM) conditions. After maintaining DRG neurons in high glucose for 1 week, we observed a significant increase in capsaicin (CAP)-evoked currents and CAP-evoked depolarizations, independent of TRPV1 channel expression. These functional changes were largely dependent on the expression of the receptor for Advanced Glycation End-products (RAGE), calcium influx, cytoplasmic ROS accumulation, PKC, and Src kinase activity. Like cultured neurons from neonates, mature neurons from adult mice also displayed a similar potentiation of CAP-evoked currents in the high glucose condition. Taken together, our data demonstrate that under the diabetic condition, DRG neurons are directly affected by elevated levels of glucose, independent of vascular or glial signals, and dependent on RAGE expression. These early cellular and molecular changes to sensory neurons *in vitro* are potential mechanisms that might contribute to sensory abnormalities that can occur in the very early stages of diabetes.

## Introduction

About one-half of patients suffering from diabetes mellitus (DM) have evidence of diabetic peripheral neuropathy (DPN). DNP is a chronic neurodegenerative condition, in which the main clinical symptoms include gradual loss of distal motor and sensory terminals, sensory disorders such as hyperalgesia and allodynia, often concurrent with a paradoxical appearance of positive symptoms such as pain or paresthesia during the progressive loss of sensory nerve fibers [1]. DPN sensory symptoms have been linked to functional, structural and biochemical abnormalities in sensory neurons, including peripheral demyelination, degeneration of myelinated sensory fibers and functionally impaired nociceptive unmyelinated C-fibers [2]. It has been proposed that sensitization of small, unmyelinated C-fibers and large A-fibers, may contribute to the sensory abnormalities that are characteristic of DPN [3–5]. DPN has a significantly negative impact on the quality of lives of diabetic patients and despite decades of research, the mechanisms underlying the development of DPN are not fully understood, thus hampering the development of advanced and effective therapies. While most of the research has concentrated on treatment and management of symptoms in long-term diabetes, there is still the need to better understand the mechanisms that trigger DPN symptoms in the early stages of the disease.

The transient receptor potential vanilloid 1 (TRPV1) is a nonselective cation channel, highly permeable to Ca^2+^ ions, expressed in primary sensory C-fibers and microglia [6,7]. Pharmacological manipulations of TRPV1 function have shown that the channel is a polymodal detector and is sensitive to a variety of stimuli, including the active component of chili peppers capsaicin, protons (pH), heat (> 42°C), and arachidonic acid [7]. TRPV1 expression level and function have been reported to be up-regulated in dorsal root ganglion (DRG), spinal dorsal horn, and the endogenous antinociceptive center of animal models of neuropathic pain [8]. The latter supports the idea that the TRPV1 channel plays an essential role in nociception. Accordingly, thermal hyperalgesia and mechanical allodynia resulting from inflammation or nerve injury models were alleviated by inhibiting TRPV1 function or by down-regulating its expression [9,10].

The influence of hyperglycemia on TRPV1 channels expressed in sensory neurons has been studied before in animal models of diabetes. Streptozocin (STZ)-induced diabetes in rodents lead to the development of DPN symptoms that are similar to those described in humans: at early stages they are manifested as mechanical allodynia, which in some cases was concomitant with thermal hyperalgesia; and at later stages they are shown as a decreased sensitivity to mechanical and thermal stimuli, and hypoalgesia [11]. In STZ-induced diabetic rodents, DRG neurons showed a potentiation of TRPV1 expression and TRPV1-mediated responses and an increase in markers of programmed cell death in both rats [12] and mice [13]. In diabetic mice, the expression level of TRPV1 in DRG neurons correlated with the magnitude of the neuropathic phenotype [13]. However, it was subsequently reported that STZ by itself had a direct effect on inducing sensory neuropathy by driving the expression of TRPV1 channels in neurons (sensory and spinal) and by activating microglia in the spinal cord, all of these effects were independent of hyperglycemia [14]. Thus, it remains unclear which factors present in the DM pathological environment contribute to the early development of the DPN-related symptoms, and particularly questions the actual effect of hyperglycemia on sensory neurons during diabetic conditions.

The dysregulation of glucose levels in DM is known to activate multiple distinct metabolic pathways leading to a singular end result—oxidative stress. For instance, increases in metabolic flux have been associated with dynamic changes in the mitochondrial morphology, which contributes to the overproduction of reactive oxygen species (ROS) in sensory neurons of the DRG [15–19]. Furthermore, ROS accumulation has been linked to the formation of advanced glycation end products (AGEs) in various diabetic tissues. AGEs are formed primarily by the non-enzymatic reaction of sugars with proteins, and they can cause random crosslinking of proteins, or more directly, they can interact with their membrane receptor, RAGE. RAGE, a member of the immunoglobulin protein family, also binds certain members of the S100/calgranulin family and pro-inflammatory proteins such as high-mobility group box 1 (HMGB1) [20]. RAGE signaling has been linked to the development of long-term complications of diabetes including neuropathy and loss of sensation/hypoalgesia [21,22]. *In vitro*, RAGE expression, signaling, and RAGE-induced ROS production contributed to apoptosis of DRG neurons exposed to high glucose conditions [23]. Still, how the RAGE-ROS pathway induces these pathological changes in sensory neurons remains obscure.

To study the effect of high glucose on sensory neurons, we performed a series of experiments designed to evaluate the currents mediated by the TRPV1 channel. Although these channels have been extensively studied as sensory transductors, their function during high glucose conditions and their possible contribution to sensory neuron abnormalities remain under investigation. Previous studies have shown that exposing cultured embryonic DRG neurons to high glucose triggers the induction of programmed cell death [24]. However, in some of those experiments, cultured neurons were maintained in media containing exceedingly high levels of glucose [24], control media contained 30 mM glucose and high glucose media contained 50 mM glucose. The extreme glucose conditions may have affected the results of the study, and thus requires further investigation using experimental conditions that are more representative of a diabetes state. In the present study, we show that capsaicin (CAP)-evoked currents were potentiated in the high glucose condition using only 25 mM glucose, and that this potentiation did not occur in neurons maintained in control condition at 5 mM glucose, or in neurons from RAGE knock-out (KO) mice maintained in the same glucose conditions. The potentiation of CAP-evoked responses in WT mice were dependent not only on RAGE expression but also on calcium entry, cytoplasmic ROS accumulation, and protein kinase activity. These results indicate that RAGE signaling is required for abnormal sensory responses, suggesting that RAGE signaling underlies the development of TRPV1-related abnormal responses in diabetes.

## Materials and methods

### Mice

A colony of RAGE knock-out (RAGE KO) mice on a C57BL/6 background was maintained by breeding heterozygous mice. Heterozygous mice were generated by back-crossing RAGE KO (homozygous) mice [25] with C57BL/6 wild type (WT) mice. All experiments were done on lumbar dorsal root ganglion (DRG) obtained from homozygous (RAGE KO) mice and their C57BL/6 (wild type) littermates. Heterozygous mice generated from our breeding strategy were solely used as breeding animals and were not used for experimental purposes in this study. Mice were genotyped using genomic DNA and polymerase chain reaction as previously described [25,26]. Most experiments were done with neonatal pups (P0–P3), except those in which DRG neurons were cultured from 2-3-month-old mice. Adult tgCGRP-eGFP mice maintained in a CD1 background, and CD1 WT mice, were generously provided by Dr. Sean Mulligan [27]. These transgenic mice, which express enhanced green fluorescence protein (eGFP) in sensory neurons under the control of the CGRP promoter, allowed us to identify CGRP+ peptidergic (nociceptive) neurons in cultures generated from adult mice.

### Cell cultures

All experiments were approved by the University of Saskatchewan’s Animal Research Ethics Board, and adhered to the Canadian Council on Animal Care guidelines for humane animal use. DRGs from neonatal C57BL/6 mice between postnatal day 1 to 3 (P1-P3) were used for the preparation of the dissociated DRG cultures. The methods used to dissociate the neurons were similar to the protocol used to dissociate superior cervical ganglion neurons [28]. Briefly, mice were euthanized by cervical transection in a sterile environment. Lumbar (L1 to L5) DRGs from the spinal cord were dissected and plated on a Petri dish with serum-containing media (L15 supplemented with vitamins, cofactors, penicillin-streptomycin and 5 mM glucose, and 10% horse serum). Small iris scissors were used to trim the remaining nerve roots from the ganglia. Once cleaned, the ganglia were submerged in Hanks balanced salt solution (HBSS) containing 0.1% trypsin (180–200 U/mL; Worthington, Freehold, NJ, USA) buffered with 1 M of HEPES (pH 7.4), and incubated at 37°C for 30–45 min in a water bath for enzymatic dissociation of the ganglia. Next, DRGs were mechanically dissociated with a fire-polished pipette. The dissociated neurons were rinsed twice with serum-containing media to inactivate trypsin. The resulting cell suspension was transferred to growth media consisting of L-15 (Gibco, Carlsbad, CA, USA) supplemented with vitamins, cofactors, penicillin-streptomycin (Invitrogen—Life Technologies, Carlsbad, CA, USA), 5% rat serum (made in house), 7S Nerve Growth Factor (10 ng/ ml; NGF; Alomone Labs, Jerusalem, Israel) and 5 mM of glucose (Sigma, St. Louis, MO, USA). The neurons were plated on laminin-coated coverslips attached to modified 35 mm tissue culture dishes. Cells were maintained at 37°C in a 95% air and 5% CO_**2**_ environment and fed every 3–4 days with growth media. To eliminate non-neuronal cells, cultures were treated with cytosine arabinoside (ARA-C; 10μM; Sigma) from day 1 to day 3. Cells were allowed to recover from stress and axotomy for one week prior to any treatment or experimental procedure. Cultured neurons were maintained in medium containing either 5 mM glucose (control) or switched to 25 mM glucose (high glucose), for an additional week. Thus, two weeks following the day of culture, cells were used in electrophysiological experiments.

Isolation of DRG neurons from adult transgenic CGRP- eGFP CD1 mice were similar to the protocol used in [29] with minor modification. Briefly, dissected lumbar DRGs were incubated at 37°C in 0.1% collagenase type 2 (Worthington, Freehold, NJ, USA) in Dulbecco’s modified eagle medium (DMEM, Gibco, Carlsbad, CA, USA) for 30 minutes and then in 0.1% trypsin (Worthington) for an additional 30 minutes. After titration in DMEM containing 10% horse serum, cells were resuspended in DMEM-containing growth media supplemented with vitamins, cofactors, penicillin-streptomycin, 5% rat serum, 7S NGF and 5 mM of glucose (as described for neonatal neurons). Maintenance of cultured adult neurons was as indicated for neonatal neurons.

To monitor for neuronal loss, we performed cell counts and detected apoptotic/necrotic cells. For cell counts we quantified cells at 4, 14 and 21 days after culturing DRG neurons from WT mice, for dishes in the high glucose condition cells were switched to 25 mM glucose at day 7 after culturing. Neurons were considered healthy by the appearance of clear nuclei and smooth cell membrane. For the detection of apoptosis/necrosis, cells were labelled with annexin V (conjugated with fluorescein isothiocyante, FITC) and propidium iodide (PI) using an apoptosis detection kit (Sigma). Annexin V and PI staining were performed following the manufacturer’s recommendations. We used cells 21 days after culturing, which corresponds to 2 weeks in the high glucose condition. Some cells were exposed to media without NGF (48 hr) as a positive control to induce cell dead. Cultures were generated from 3 different platings, each from 10 neonate mice. Images were collected with the help of an AxioObserver inverted microscope and Zen software (Carl Zeiss, Oberkochen, Germany).

### ROS measurements

To measure cytoplasmic ROS levels, we used the redox- sensitive dye CM-H_**2**_DCFDA (Invitrogen, Burlington, Ontario, Canada), an acetoxymethyl (AM) ester. We used cultured DRG neurons from wild-type or RAGE-KO mice maintained in either control or high glucose for 1–2 weeks. Cultures were incubated for one hour at 37°C with medium containing CM-H_**2**_DCFDA (10μM) and subsequently washed five times with standard extracellular solution (see below). The cultures were then placed on the stage of an inverted epifluorescence microscope (AxioObserver Z1) and viewed through 40x oil-immersion objective (Carl Zeiss) at room temperature. Data were acquired with the help of either Axiovision or Zen software (Carl Zeiss) and analyzed with ImageJ (NIH, Bethesda, MD, USA) software. For each image, we defined regions of interest (neuronal cell body, excluding the nucleus) and measured the mean fluorescent intensity. The averaged control fluorescence intensity, *F*_*C*_, (minus the background), was determined from neurons maintained in control levels of glucose. For each neuron maintained in high glucose, we obtained its mean fluorescent intensity (minus background), *F*_*HG*_. Lastly, the fluorescent intensity for each neuron in high glucose was normalized to the mean control fluorescent intensity and expressed as *F*_*HG*_*/F*_*C*_. A total of 596 neurons were measured from cultures obtained from both, wild-type (163 neurons in control and 102 neurons in high glucose) and RAGE-KO (174 neurons in control and 157 neurons in high glucose) mice. Three independent neuronal platings from approximately 10 pups each, were used for wild type and RAGE-KO mice.

### Patch-clamp electrophysiology

CAP-evoked currents were recorded using the whole-cell patch-clamp technique [30]. Patch pipettes were made from borosilicate glass (WPI, Sarasota, FL, USA) glass using a vertical puller (PC 10; Narishige Scientific Instrument Lab., Tokyo, Japan) and were fire-polished with a microforge (MF 900; Narishige). Micropipettes had a resistance of 5–10 MΩ when filled with intracellular recording solution, and formed gigaseals of 1–8 GΩ. Recording electrodes were filled with the following solution (in mM, all from Sigma): 60 KAc, 70 KF, 5 NaCl, 1 MgCl_**2**_, 1 CaCl_**2**_, 2 MgATP, 10 EGTA, and 10 HEPES, and pH was adjusted to 7.2 with KOH. For experiments using antioxidants, 500 μM of the antioxidant α-lipoic acid (ALA; Sigma) and 1,000 U/mL of catalase (CAT; Sigma) was prepared fresh on the day of the experiment and dissolved in intracellular solution. The selective PKC inhibitor LY333531 ((9S)-9-[(Dimethylamino)methyl]-6,7,10,11-tetrahydro-9H,18H-5,21:12,17-dimethenodibenzo[e,k]pyrrolo[3,4-h][1,4,13]oxadiazacyclohexadecine-18,20(19H)-dione hydrochloride) (50 nM; Sigma) was added to the intracellular solution. Once the whole-cell configuration was achieved, the intracellular solution was allowed to dialyze the cell for 15 min prior to starting the recordings [28]. For experiments using the Src kinase inhibitor PP2 (4-amino-5-(4-chloro- phenyl)-7-(t-butyl)pyrazolo[3,4-d]pyrimidine) (20 μM; Sigma), cells were incubated for 30 min before electrophysiological recordings. The external solution contained (in mM): 140 NaCl, 5.4 KCl, 0.33 NaH_**2**_PO_**4**_, 0.44 KH_**2**_PO_**4**_, 1 MgCl_**2**_, 1 CaCl_**2**_, 10 HEPES, and 5 glucose, and pH was adjusted to 7.4 with NaOH. For barium experiments, 1 mM of BaCl_**2**_ was used in place of CaCl_**2**_ in the external solution. Whole-cell currents or membrane potentials were recorded at room temperature with the aid of an Axopatch 200B amplifier (Molecular Devices, Palo Alto, CA, USA) equipped with a 1 GΩ headstage feedback resistor, and sampled at 1kHz and 5 kHz for ATP and CAP experiments respectively, with a Digidata 1440A. Voltage clamp protocols, data acquisition and analysis were performed using pCLAMP 10 software (Molecular Devices) and Origin 7 (OriginLab Coorporation, Northampton, MA, USA). Once the whole-cell configuration was achieved, cells were allowed to stabilize for 5 min before recording. Control and CAP extracellular solutions were applied using a pressurized perfusion fast-step system (SF-77B, Warner Instruments, Hamden, CT, USA) that provided a constant flow of 2–3 ml/min. Tetrotodoxin (TTX, 1 μM) was added to both control and CAP solutions to prevent space-clamp artifacts during whole-cell recording.

### Western blotting and immunocytochemistry

For western blotting, suspensions of DRG neurons from WT and RAGE KO mice were prepared as described above, and plated onto specialized cell culture dishes (Sarstedt, Nümbrecht, Germany). We used 3 replicas per condition (control or high glucose), for both WT and RAGE KO mice. Each replica was generated from 10 pups. Neurons were lysed using a 1% NP-40 lysis buffer containing protease inhibitor cocktail. Protein concentration was determined by the Bradford assay. Samples were resolved using 10% SDS-PAGE and then electrotransferred onto a nitrocellulose membrane (Bio-Rad Laboratories, Hercules, CA, USA). The membrane was then incubated with the following primary antibodies overnight at 4°C: mouse anti-mouse β-actin horse radish peroxidase(HRP)-conjugated (1:1000; Santa Cruz Biotechnology, Santa Cruz, CA, USA), and rabbit anti-mouse TRPV1 (1:1000; Alomone Labs). Each membrane was then washed three times with TBST (20 mM Tris, 150 mM NaCl, 0.1% Tween 20) before being probed with a HRP-conjugated goat anti-rabbit secondary antibody (1:20000; Bio-Rad Laboratories). Protein signals were visualized using enhanced chemiluminescence reagents (Bio-Rad) and quantified by densitometry using ImageJ software (NIH).

For immunocytochemistry, cultured neurons were fixed with 4% paraformaldehyde for 1 hour at room temperature [28]. After washing with PBS (3 times for 3 min), the cells were incubated overnight at 4°C with a rabbit anti-TRPV1 (1:1000, Alomone Labs) diluted in PBS containing 1% BSA and 0.5% Triton X-100. The next day, the samples were washed in PBS (3 x 3 min) and then incubated in the dark for 1 hour at room temperature with a goat anti-rabbit secondary antibody conjugated to FITC (1:500; Invitrogen—Thermofisher, Carlsbad, CA, USA). Samples were covered with an anti-photobleaching reagent (Vectashield; Vector Laboratories, Burlingame, CA, USA) and viewed with an epifluorescence microscope (AxioObserved; Zeiss). Fluorescence intensity was quantified as the difference in intensity of the region of interest (neuronal cell body, excluding the nucleus) to the background.

### Statistical analysis

To compare two means, we used parametric *t*-test or non-parametric Mann-Whitney U test. For the comparison of three or more means we used ANOVA (one-way or two-way, as specified in figure legends), followed by multiple comparisons post-hoc tests. For the comparisons of means over time we used repeated measurement one-way ANOVA followed by Tukey post-hoc test. Non-parametric analysis was used for groups of means that did not follow a normal distribution. All statistical analyses were done using Graphpad InStat 3.0 or Prism 6 software (GraphPad Software Inc., La Jolla, CA, USA). All values are reported as mean ± S.E.M. and p < 0.05 as criterion for significance.

This work was approved by the University of Saskatchewan’s Animal Research Ethics Board (Campanucci: protocol 20090082) and adhered to the Canadian Council on Animal Care guidelines for humane animal use.

## Results

### High glucose potentiates CAP-evoked responses in DRG neurons

**Fig 1:**
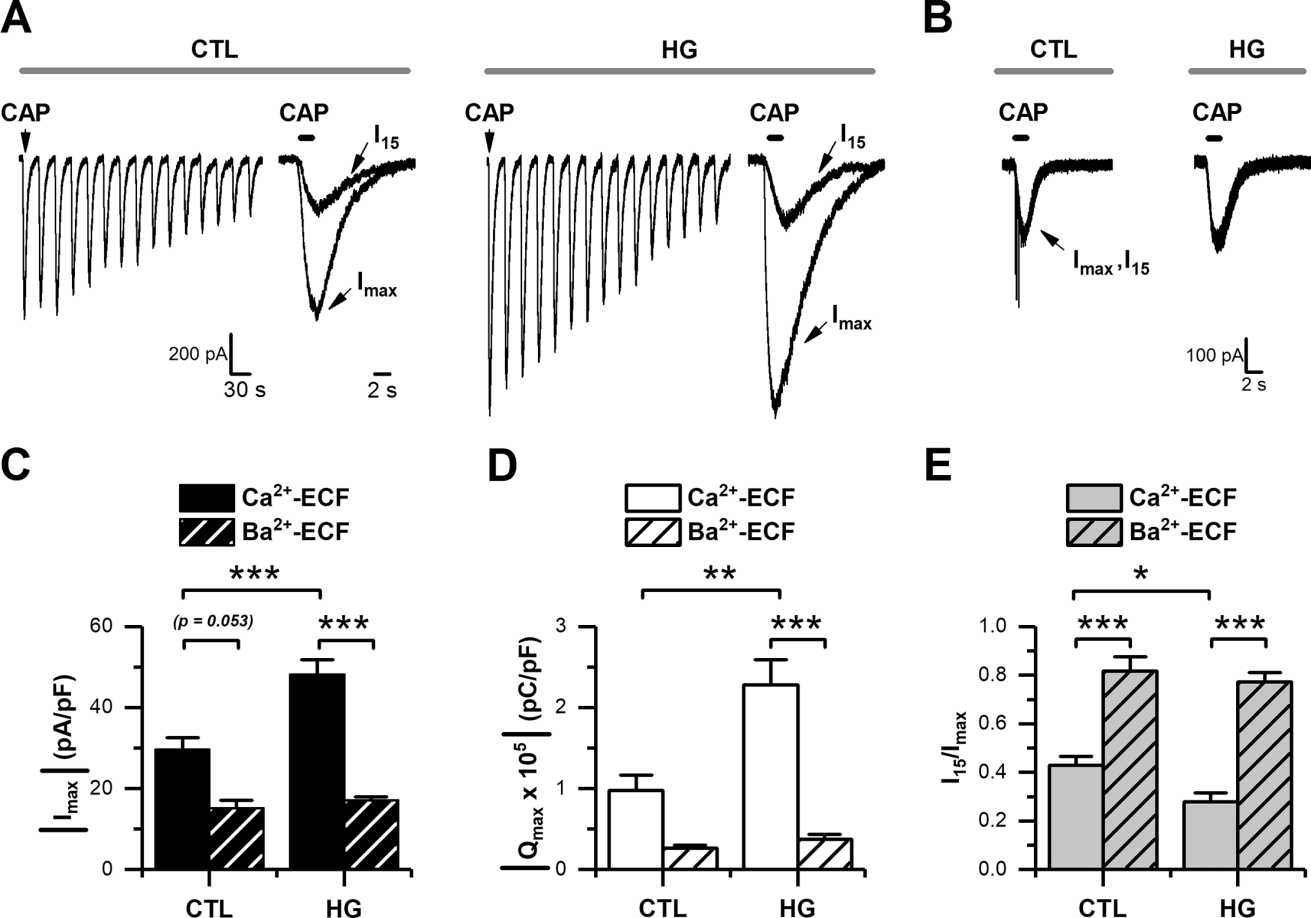
High glucose potentiated CAP-evoked currents in cultured DRG neurons. A-B, representative traces of CAP-evoked currents recorded in whole-cell voltage-clamp mode (holding potential set at -60 mV). Agonist was applied for 1 s and repeated every 30 s, in control (CTL), and high glucose (HG) in Ca^2+^-ECF (A), and in the same glucose conditions but in Ba^2+^-ECF (B), respectively. C-E, bar graphs summarizing maximal current (I_max_) density (C), maximal current charge (Q_max_; D), and maximal current rundown calculated as the ratio of I_max_ relative to the 15^th^ application (I_15_) in a series (E), for CTL (n = 24) and HG (n = 24) in Ca^2+^-ECF; and for CTL (n = 8) and HG (n = 11) in Ba^2+^-ECF, respectively. All data are represented as mean ± SEM. The absolute current density and charge were used in C and D for simplicity. Statistical analysis by two-way ANOVA followed by Sidak's post-hoc test. *, p < 0.05; **, p < 0.01; ***, p < 0.001.

To study the effect of high glucose on sensory neurons, we performed a series of experiments designed to evaluate the currents mediated by the transient receptor potential 1 (TRPV1). Neonatal mouse DRG neurons were maintained in either 5 mM of glucose (control) or 25mM of glucose (high glucose) for 1 week [26,28]. Under these experimental conditions, we used repetitive applications of the TRPV1 agonist capsaicin (CAP, 5 μM, at 30 s intervals), which normally causes a use-dependent rundown of CAP-evoked currents in the presence of Ca^2+^ ions (Fig 1A) [31]. We examined the maximal peak current density (I_max_) and charge carried by the maximal current (Q_max_), and both revealed a significant increase in the 25 mM glucose condition (Fig 1C and 1D). To evaluate the use-dependent rundown of CAP-evoked currents, we calculated the ratio between the 15^th^ current in a series (I_15_) and the maximal peak current (I_max_). The analysis of the ratio I_15_/I_max_ showed that in the control condition there is approximately 40% of CAP-evoked current remaining after the 15^th^ application of the agonist. However, there is only approximately 27% of current remaining during high glucose conditions (Fig 1E).

To evaluate both, the contribution of Ca^2+^ ions carried by TRPV1 channels to the potentiation of CAP-evoked responses, and their role in driving the use-dependent rundown of TRPV1 currents over repetitive applications of CAP [32,33], we repeated the experiments substituting Ca^2+^ with the divalent cation barium (Ba^2+^; Ba^2+^-ECF) in the extracellular fluid (ECF). As expected for an ion channel highly permeable to Ca^2+^, using Ba^2+^-ECF caused a non-significant reduction in the basal CAP-evoked current recorded in the control condition (Fig 1B), and more importantly, a lack of potentiation of CAP-evoked currents in high glucose (Fig 1B–1D). Lastly, we observed that CAP-evoked currents recorded in Ba^2+^-ECF were markedly stable in both experimental conditions, maintaining approximately 85% of the I_15_/I_max_ ratio (Fig 1E). Collectively, these findings suggest that an increase in Ca^2+^ influx under the high glucose condition is the source for the potentiation of CAP-evoked currents and use-dependent rundown of TRPV1 currents.

**Fig 2:**
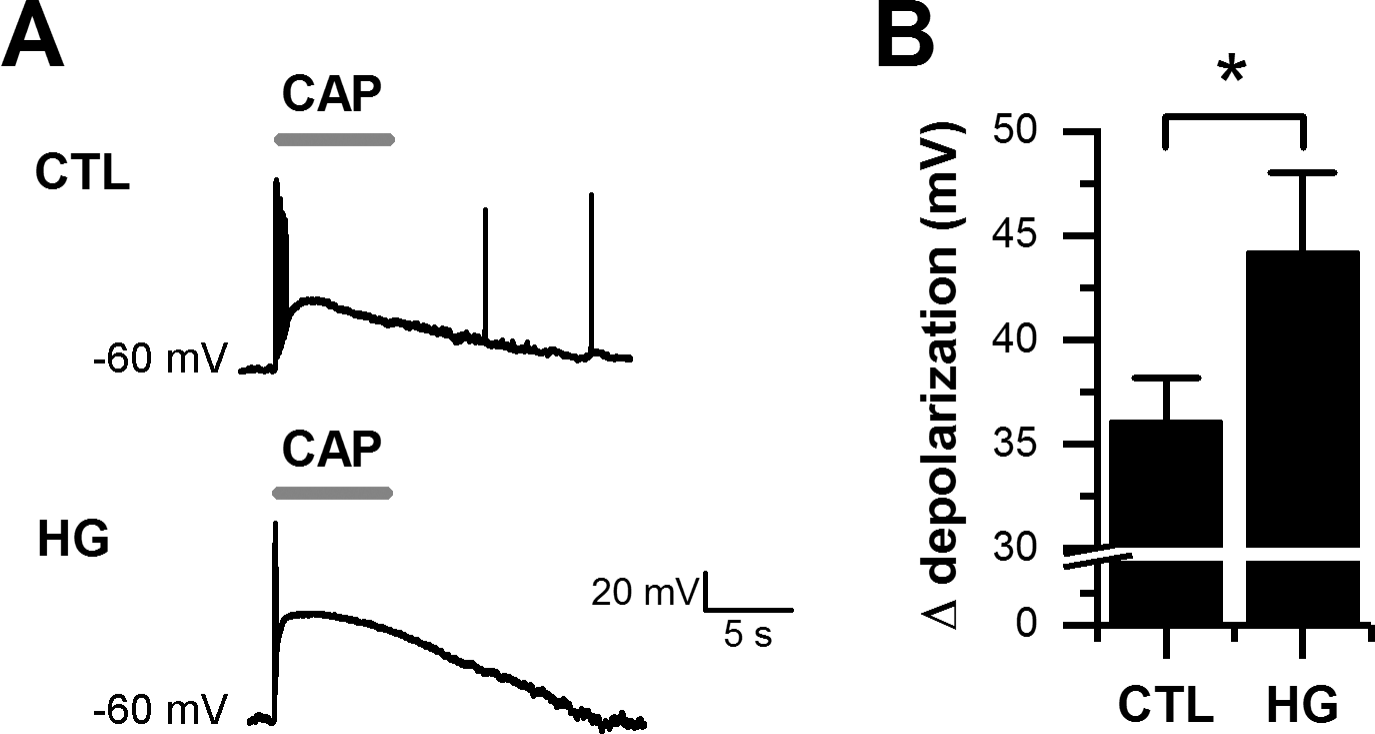
High glucose enhanced CAP-evoked depolarizations in cultured DRG neurons. A, representative traces showing membrane depolarization induced in a single neuron from control (CTL, top trace) or high glucose (HG, bottom trace) condition by 5 s applications of CAP during whole-cell current clamp recordings. B, the bar graph summarizes the change in membrane potential evoked by the application of CAP. All data are represented as mean ± SEM, n = 8 for each condition. Statistical analysis by unpaired *t*-test; *, p < 0.05.

**Fig 3:**
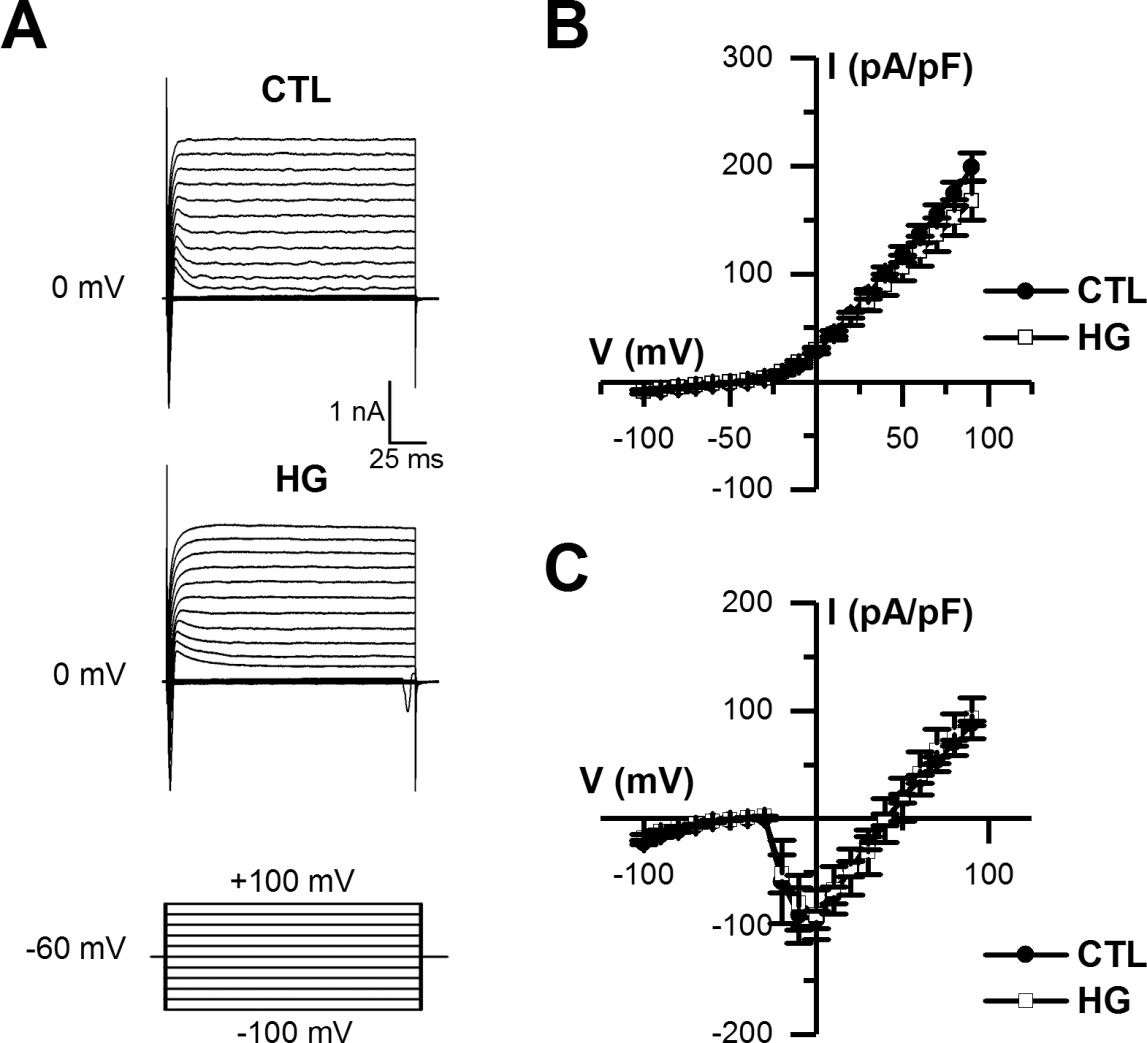
Voltage-gated currents were unaffected by high glucose conditions. A, show representative traces of macroscopic voltage-gated outward and inward currents recorded in voltage-clamp mode (holding potential set at -60 mV) by the application of a voltage step protocol (inset), in cultured DRG neurons maintained in either control (CTL, top traces) or high glucose (HG, bottom traces). B-C, the I-V plot summarize the mean outward current density (B) and mean inward peak current density (C) obtained at different step voltages, in both experimental conditions. All data are represented as mean ± SEM, n = 5 for each condition.

To study the effect of high glucose on CAP-induced depolarizations, we applied CAP (5 s) under current-clamp mode and quantified the change in membrane potential (Δ depolarization). Before the application of CAP, the cell membrane potential was maintained at -60 mV by current injection to mitigate the effects of differences in resting potentials between treatment groups. We observed a stronger depolarization when cells were maintained in 25 mM glucose (Fig 2A and 2B). These observed changes in electrophysiological properties of CAP-evoked responses were not accompanied by significant changes in passive membrane properties, quantified as resting potential (*V*_*m*_), input resistance (*R*_*in*_) and membrane capacitance (*C*_*m*_) (Table 1). Furthermore, no changes were observed in voltage-gated Na^+^ or K^+^ currents when compared between control and high glucose conditions (Fig 3). Collectively, we report that elevating glucose levels (to 25 mM) for one week was sufficient to induce changes in TRPV1 channel function, and consequently greater cell depolarization following exposure to CAP.

**Table 1:** Passive membrane properties for cultured DRG neurons from wild type mice.

	*V*_*m*_ (mV)	*R*_*in*_ (MΩ)	*C*_*m*_ (pF)	*n*
**CTL**	*-*43.03 ± 1.17	28.20 ± 2.12	16.69 ± 0.91	26
**HG**	-41.33 ± 1.16	25.80 ± 1.74	18.27 ± 0.77	24

Table summarizes the membrane potential (*V*_*m*_), input resistance (*R*_*in*_), and cell capacitance (*C*_*m*_) obtained during whole-cell recording of cultured DRG neurons from WT mice maintained in either control or high glucose conditions. Means were compared by unpaired *t*-test, no significant differences were found between the treatments.

**Fig 4:**
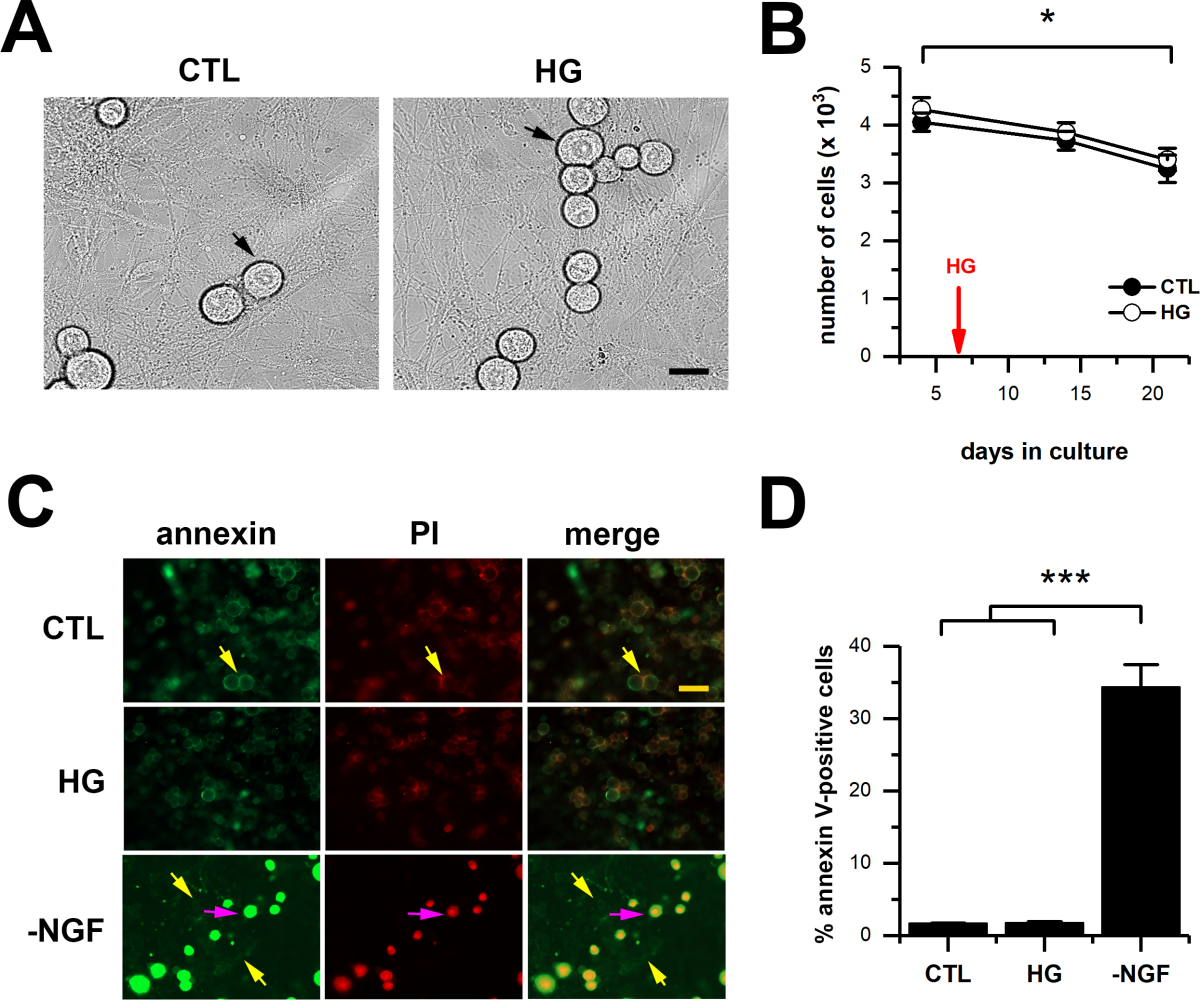
DRG neuronal counts for cultures from WT mice, maintained in either control (CTL) or high glucose (HG). A, representative phase-contrast images of neurons at 21 days after culturing, maintained in CTL or HG (for 14 days), show healthy neurons with clear nuclei (arrows) and smooth membrane (40x magnification). B, bar graph summarizes the number of neurons per culture dish in CTL (n = 7) and HG (n = 7) at 4, 14 and 21 days after culturing. All cultures were generated in control levels of glucose and half of them where switched to high glucose levels at 7 days after culturing (red arrow). C, representative images for annexinV-FITC and propidium iodide (PI) fluorescence, in either CTL, HG, or in -NGF as positive control (20x magnification). Yellow arrows show representative examples of non-apoptotic cells, and purple arrows show an example of an apoptotic/necrotic cell. D, bar graph summarizes the percentage of annexinV-positive cells (n = 4, per condition), 1.6% for CTL, 1.7% for HG, and 34.3% for -NGF. All data are represented as mean ± SEM, means were compared by repeated measures one-way ANOVA followed by Tukey post-hoc test (B); and by one-way (non-parametric) ANOVA followed by Dunn’s post-hoc test (D). *, p < 0.05. Scale bars represent 30 μm (A) and 50 μm (C).

Since previous reports revealed that exposure of embryonic DRG neurons to high glucose induced neuronal loss [24,34], we monitored cell viability during the length of our experiments. We performed neuronal counts for up to 21 days after culturing, and 2 weeks after cells were switched to the high glucose condition. Our data showed that there were no significant differences in the number of cells between treatments at 14 and 21 days after culturing (Fig 4). We only observed a significant decreased of approximately 10% in the number of cells between day 4 and day 21, which was independent of the treatment, and which is within the acceptable range for neurons maintained in culture conditions for 3 weeks. In addition, to determine whether the exposure to the high glucose treatment caused neuronal loss due to toxicity and/or triggered apoptotic cell death, we use the detection of propidium iodide (PI) and annexin V markers. We found that in control and high glucose conditions, only 1.6% and 1.7% of the cells were annexin V and/or PI positive, respectively (Fig 4C and 4D). In contrast, when NGF deprivation was used as a positive control to induce neuronal loss [35,36], we observed a 34.3% of annexin V / PI positive cells (Fig 4C and 4D). Therefore, our data confirms that exposing neonatal cultured DRG neurons to 25 mM glucose for up to 2 weeks does not induce neuronal loss.

### High glucose fails to potentiate CAP-evoked currents in DRG neurons from RAGE KO mice

Increased RAGE expression has been previously reported in the DRGs of long-term diabetic rats [37], and in the development of neuropathic pain after nerve injury [38]. However, whether RAGE expression is linked to the electrophysiological abnormalities we observed with CAP-evoked responses under high glucose in this study, remains to be explored.

**Fig 5:**
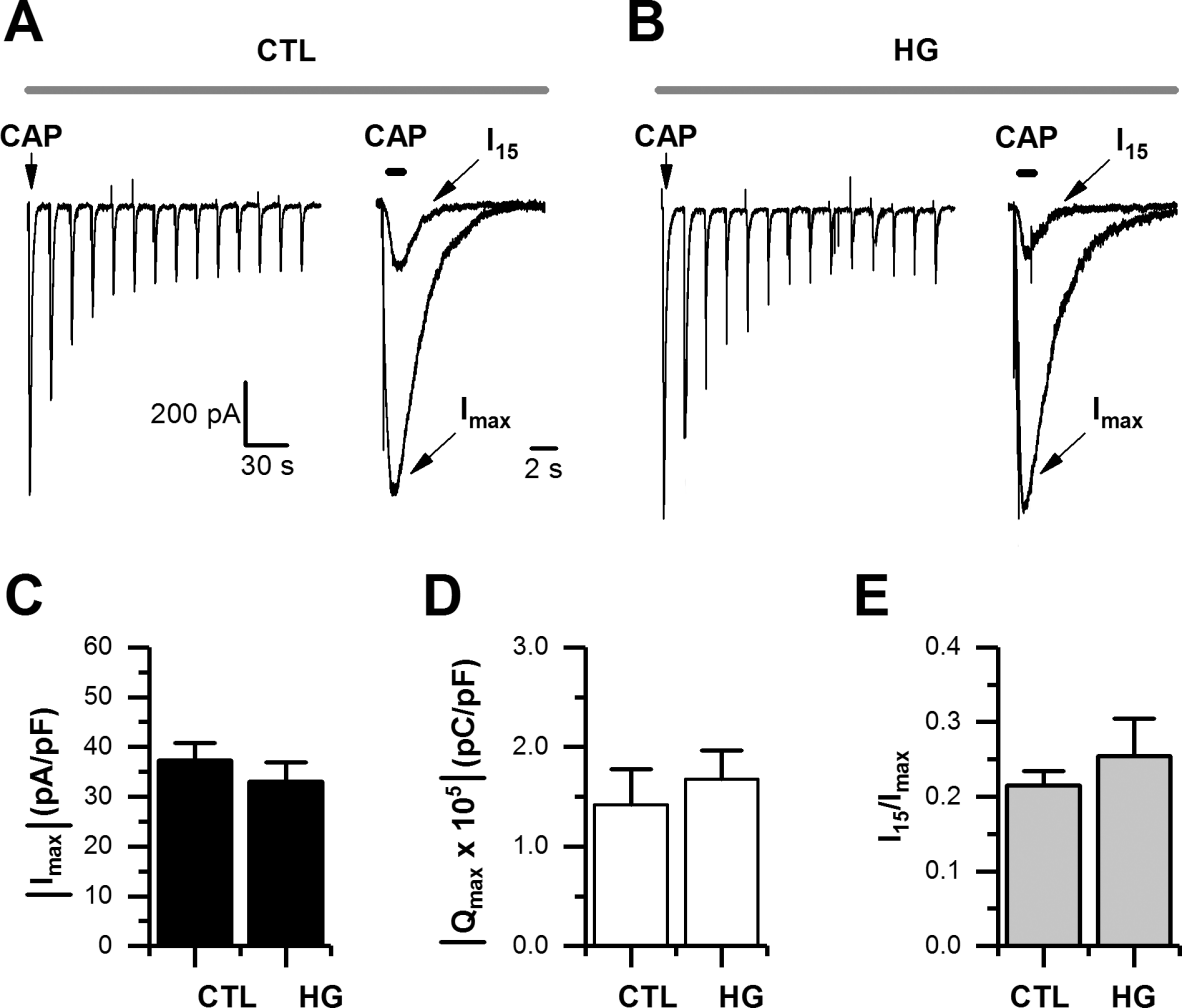
High glucose failed to potentiate CAP-evoked currents in cultured DRG neurons from RAGE KO mice. A-B, representative traces of CAP-evoked currents recorded during whole-cell voltage-clamp recording (holding potential set at -60 mV) from DRG neurons maintained in either control (CTL) or high glucose (HG) conditions. C-E, bar graphs summarize maximal current (I_max_) density (C), maximal current charge (Q_max_; D), and current rundown calculated as the ratio I_15_/ I_max_ (E). All data are represented as mean ± SEM, n = 14 for each condition. The absolute current density and charge were used in C and D for simplicity. Statistical analysis by unpaired *t*-test (C-D), and Mann-Whitney test (E); no significant differences between treatments were found.

To test the potential role of RAGE expression on CAP-evoked currents, we cultured lumbar DRG neurons from RAGE KO mice. In a previous study by Chandna et al. [26] we worked with this particular transgenic mouse [25], and confirmed the lack of RAGE expression in various tissues, including the peripheral nervous system and lung. Cultured DRG neurons from RAGE KO mice had passive membrane properties (Table 2) and CAP-evoked currents that were indistinguishable from those obtained from WT mice (Fig 1 vs. Fig 5). Exposure of DRG neurons from RAGE KO mice to 25 mM glucose for up to 2 weeks did not result in the potentiation of CAP-evoked currents in any of the three quantified current parameters (I_max_, Q_max_, and I_15_/I_max_; Fig 5A–5E), compared to neurons from WT mice (Fig 1). These results complement our previous report for autonomic neurons [26], and demonstrates that RAGE expression is also required to mediate the electrophysiological abnormalities caused by high glucose in lumbar DRG neurons.

**Table 2:** Passive membrane properties for cultured DRG neurons from RAGE KO mice.

	*V*_*m*_ (mV)	*R*_*in*_ (MΩ)	*C*_*m*_ (pF)	*n*
**CTL**	*-*41.63 ± 2.17	26.61 ± 1.36	23.62 ± 1.05	13
**HG**	-40.59 ± 1.56	24.63 ± 1.80	23.85 ± 1.57	13

Table summarizes the membrane potential (*V*_*m*_), input resistance (*R*_*in*_), and cell capacitance (*C*_*m*_) obtained during whole-cell recording of cultured DRG neurons from RAGE KO mice maintained in either control or high glucose conditions. Means were compared by unpaired *t*-test, no significant differences were found between the treatments.

### High glucose-induced oxidative stress potentiates CAP-evoked currents in DRG neurons

**Fig 6:**
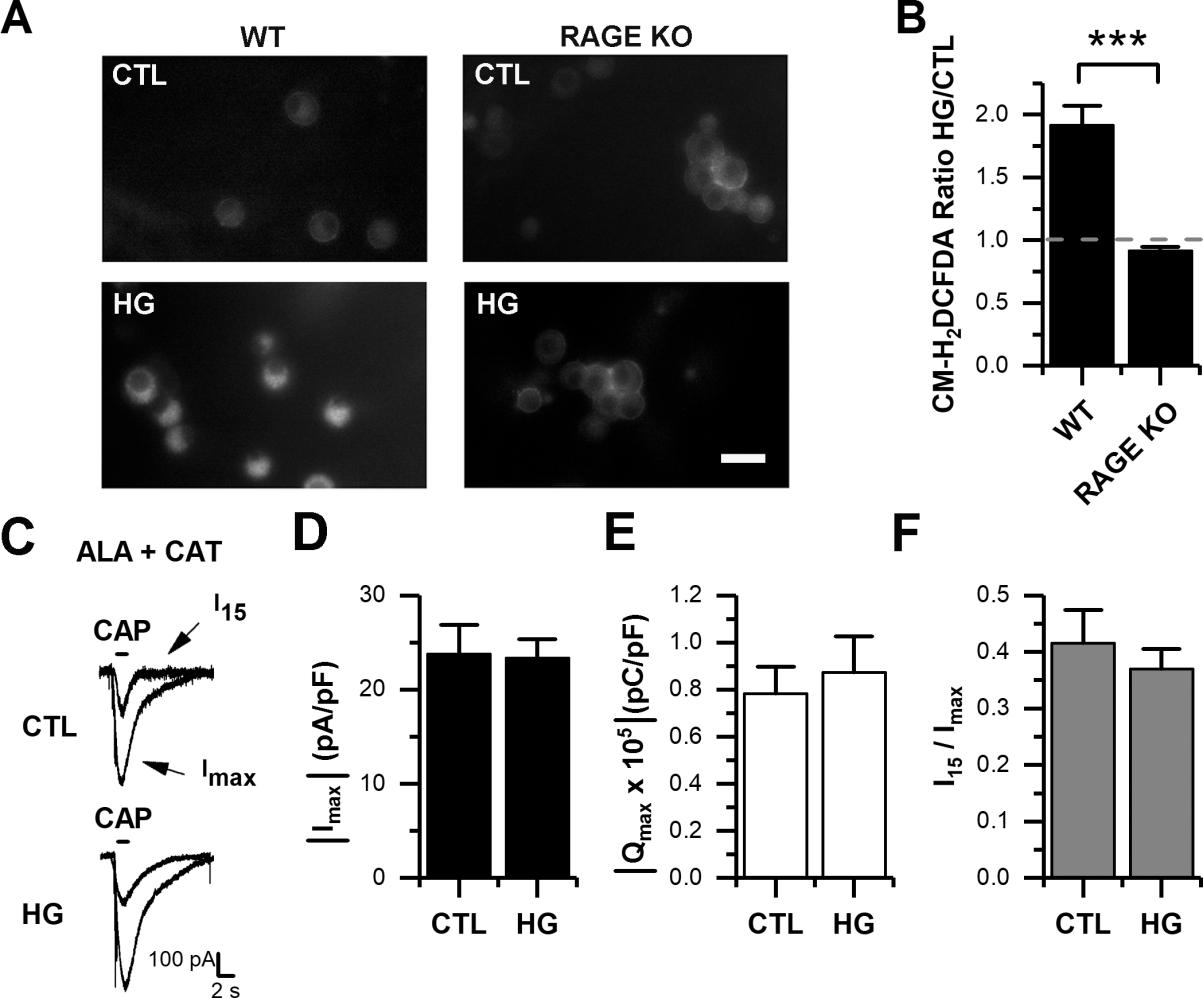
High glucose induces intracellular ROS accumulation. A, representative images of ROS detection by CM-H2DCFDA fluorescence, in cultured DRG neurons from WT and RAGE KO mice, maintained in control (CTL) and high glucose (HG) conditions for 1 week. B, the bar graph summarizes mean ± SEM pixel intensity for HG/CTL ratio for neurons from WT (n = 110) and RAGE KO (n = 156) mice, respectively. C, representative traces showing CAP-evoked currents recorded in the presence of ALA+CAT in CTL and HG conditions. D-F, bar graphs summarize maximal current (I_max_) density (D), maximal current charge (Q_max_; E), and current rundown calculated as the ratio I_15_/ I_max_ (F). All data are represented as mean ± SEM, ALA + CAT (CTL, n = 10; HG, n = 12). Scale bar in A represent 30 μm. Statistical analysis by one sample *t*-test hypothetical mean = 1 (B), unpaired *t*-test (D-E), and Mann-Whitney test (F). ***, p < 0.001.

The activation of RAGE signaling by elevated glucose has been linked by us [26] and others [23,39–41] to the generation of oxidative stress. Therefore, to understand the relationship between high glucose, RAGE and the potentiation of CAP-evoked currents in DRG neurons, we evaluated the potential involvement of oxidative stress. We monitored the cytoplasmic redox state with the ROS sensitive dye CM-H_2_DCFDA, as we have previously described [26,28], in cultured neurons from WT and RAGE KO mice maintained in either under control or high glucose. Under these experimental conditions, we observed an approximately two-fold increase (Fig 6A and 6B; p < 0.0001) in the CM-H_2_DCFDA fluorescence in neurons from WT mice exposed to 25 mM of glucose (n = 102) relative to control (Fig 6A and 6B). In contrast, we observed no significant changes in the CM-H_2_DCFDA fluorescence in neurons from RAGE KO mice (Fig 6A and 6B). We next asked whether cytoplasmic ROS accumulated during high glucose induced the potentiation of CAP-evoked currents. Thus, we repeated our whole-cell experiments in neurons from WT mice with the addition of antioxidants, α-lipoic acid (100 μM, ALA) and catalase (1,000 units/ml, CAT), in the pipette solution. ALA+CAT had no effects on the basal CAP-evoked currents (I_max_, Q_max_, and I_15_/I_max_) recorded (compare to Fig 1C and 1D). However, the use of ALA+CAT in the patch pipette prevented the potentiation of CAP-evoked currents in the high glucose condition (Fig 6C–6F). Thus, our data using solutions containing antioxidants indicate that the potentiation of CAP-evoked responses is dependent on the increased levels of cytoplasmic ROS.

### The potentiation of CAP-evoked currents during high glucose is mediated by PKC and Src kinases

**Fig 7:**
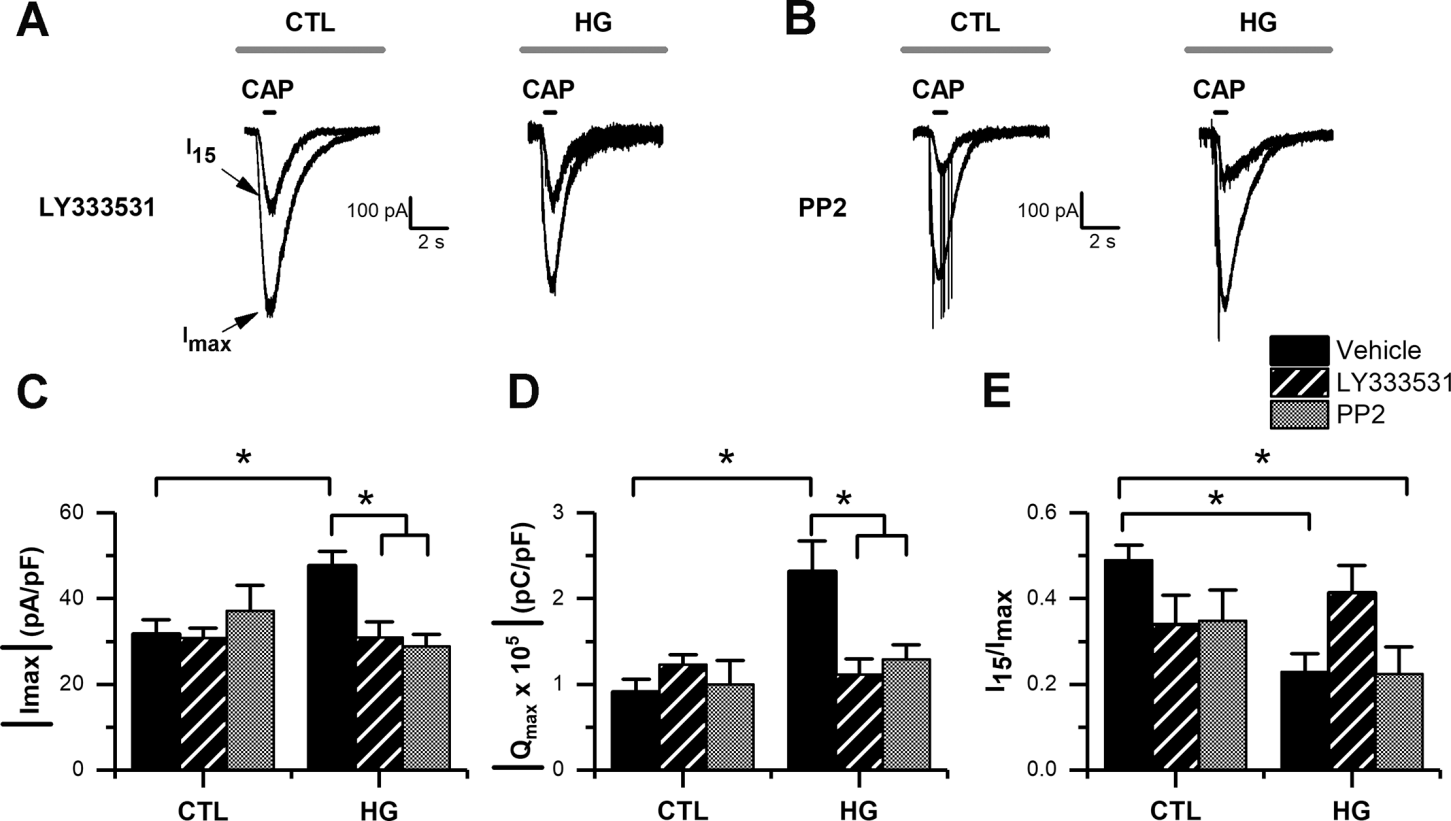
Potentiation of CAP-evoked currents was dependent on PKC and Src kinase activity. A, representative current traces of CAP-evoked currents obtained from cultured DRG neurons during whole-cell voltage-clamp recording (holding potential set at -60 mV), in control (CTL) and high glucose (HG) conditions in the presence of LY333531 and PP2. B-D, bar graphs summarize maximal current (I_max_) density (B), maximal current charge (Q_max_; C), and current rundown calculated as the ratio I_15_/I_max_ (D). Bars show treatment conditions examined: vehicle (DMSO 1μl/ml, CTL and HG n = 10), LY333531 (CTL, n = 11; HG, n = 9), and PP2 (CTL, n = 5; HG, n = 5). All data are represented as mean ± SEM. The absolute current density and charge were used in B and C for simplicity. Statistical analysis by two-way ANOVA followed by Sidak's post-hoc test. *, p < 0.05.

Next, to identify the cellular mechanisms activated under high glucose-oxidative stress conditions, we evaluated the contribution of protein kinases to the potentiation of CAP-evoked currents. It has been previously reported that PKC, particularly PKCβ [42], can sensitize TRPV1 channels, causing a gradual increase in currents upon repetitive agonist applications [42–45]. To test whether PKCβ was involved in the observed potentiation of CAP-evoked currents, we treated DRG neurons intracellularly with the PKCβ inhibitor, LY333531. LY333531 (50 nM) did not exhibit any effect on the basal CAP-evoked currents recorded in the control condition in the presence of the vehicle DMSO (1 μl/ml) (Fig 7C–7E). However, it prevented the potentiation of CAP-evoked currents in the high glucose condition (Fig 7A and 7C–7E). Finally, we tested the possible involvement of Src kinases, which potentiate TRPV1-mediated currents in colonic DRG neurons [46], and can induce rapid movement of TRPV1 channels to the surface membrane in HEK293 cells [47]. To test this possibility we incubated neurons in the selective inhibitor of the Src family kinases PP2 [48]. PP2 (20 μM) did not have any significant effect on basal CAP-evoked currents recorded in the control condition in the presence of the vehicle DMSO (1 μl/ml) (Fig 7C–7E, e). However, incubating DRG neurons with PP2 prevented high glucose-induced potentiation of CAP-evoked currents (Fig 7B and 7C–7E). Taken together, our data indicate that PKC and Src kinase signaling are responsible for the potentiation of TRPV1-mediated currents during high glucose.

### High glucose does not increase TRPV1 expression

**Fig 8:**
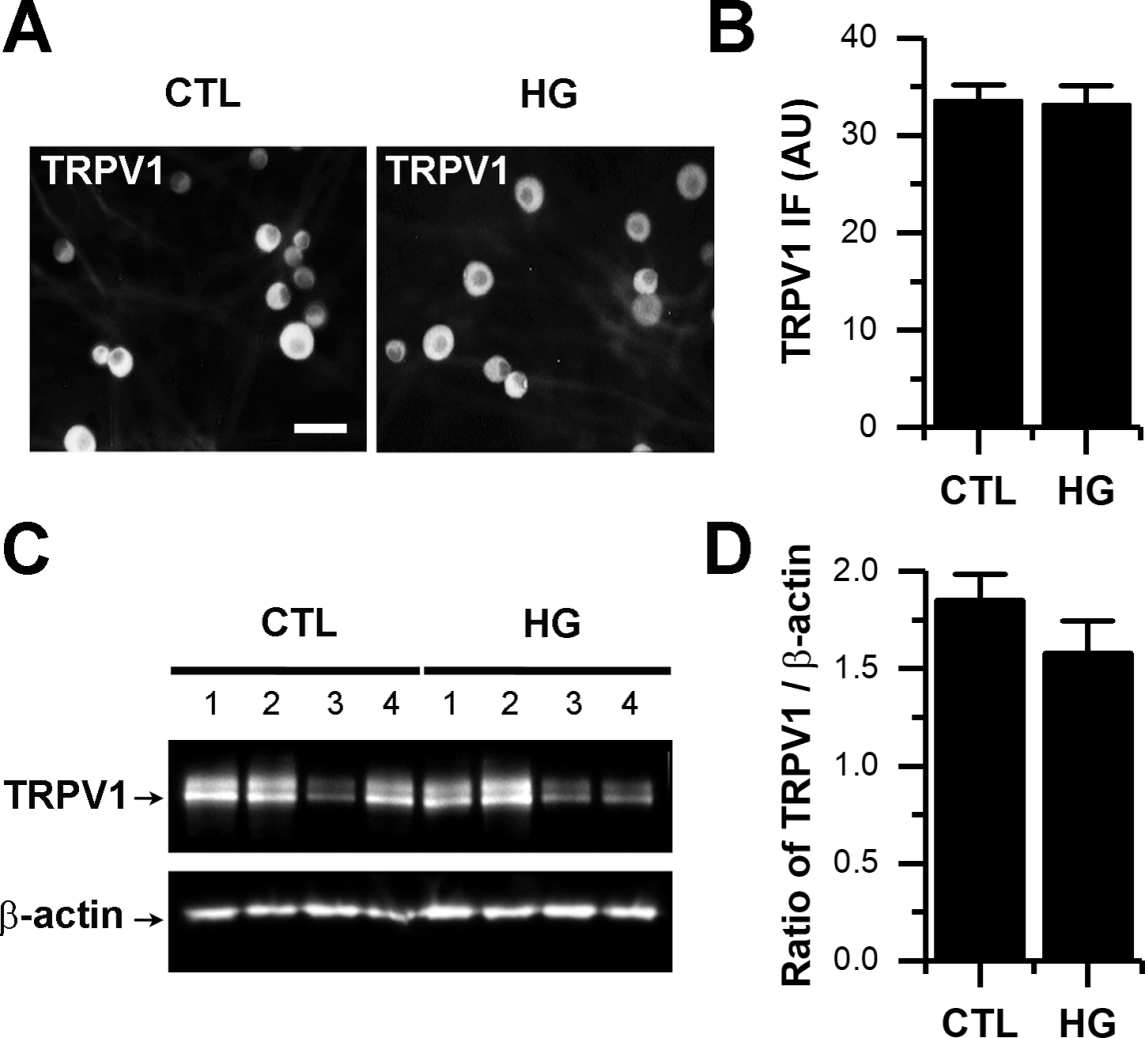
High glucose did not increase expression of TRPV1 channels. A, representative images immunocytochemistry for the detection of TRPV1 protein in cultured DRG maintained in either control (CTL) or high glucose (HG) conditions. B, bar graph summarizes mean pixel intensity for CTL (n = 71; HG, n = 51). C, western blots showing TRPV1 and β-actin expression in 4 independent samples (per treatment) of cultured DRG neurons exposed to either CTL or HG for 10 days. B, bar graph summarizes the ratio of TRPV1/β-actin per treatment. All data are represented as mean ± SEM. Scale bar in A represents 30 μm. Statistical analysis by Mann*–*Whitney test; no significant differences were found between treatments (B-D). AU, arbitrary units.

It has been previously shown that the chemical induction of diabetes by STZ was increased TRPV1 expression [14], but whether channel expression is altered in diabetes, particularly in early stages of the disease, remains unclear. Thus, we next asked if the potentiation observed in CAP-evoked currents during the high glucose condition was indeed due to increased TRPV1 expression. We quantified TRPV1 levels by immunocytochemistry and western blotting. Quantification of TRPV1 expression levels from cultures maintained in either control or high glucose conditions show no significant changes in TRPV1 expression after 10–14 days in culture (Fig 8). This suggests that, at least in the early stages of hyperglycemia, the sensitization of sensory neurons do not depend on the increased expression of TRPV1 channels but rather on post-transcriptional modification involving ROS, PKC and Src kinases.

### High glucose conditions induce potentiation of CAP- evoked currents in cultured DRG neurons from adult mice

**Fig 9:**
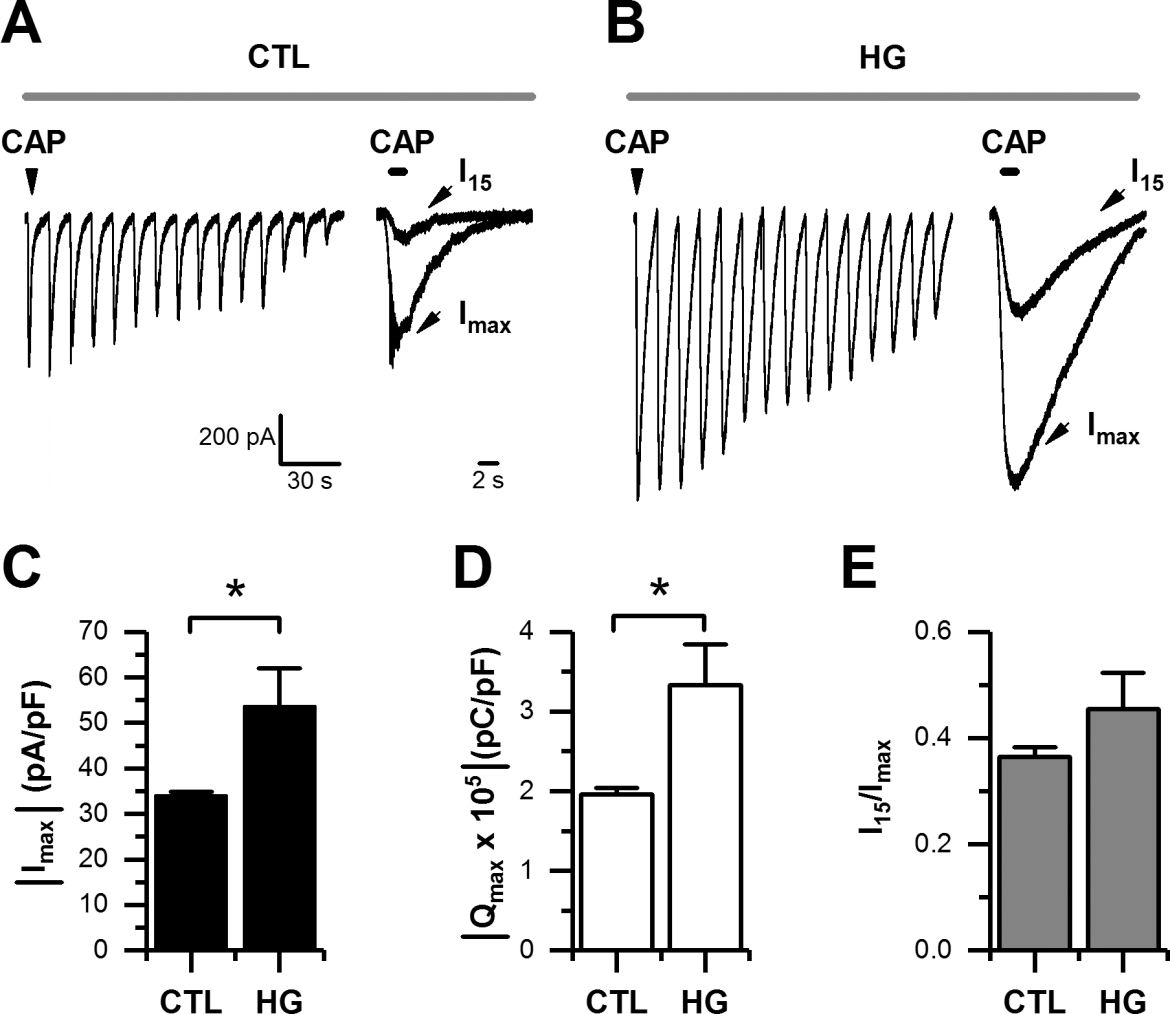
High glucose also potentiated CAP-evoked currents in cultured DRG neurons from adult mice. A-B, representative traces of CAP-evoked currents from cultured adult DRG neurons during whole-cell voltage-clamp recording (holding potential set at -60 mV), in control (CTL) and high glucose (HG) conditions. C-E, bar graphs summarize maximal current (I_max_) density (C), maximal current charge (Q_max_; D), and maximal current rundown calculated as the ratio I_15_/I_max_ (E), for CTL (n = 9) and HG (n = 8) conditions, respectively. All data are represented as mean ± SEM. The absolute current density and charge were used in C and D for simplicity. Statistical analysis by unpaired *t*-test (C-D), and Mann-Whitney test (E). *, p < 0.05.

Our experiments thus far have been performed using cultures of lumbar DRG neurons extracted from neonate (P0-P3) mice, which provide a better yield of primary cultured neurons. To evaluate the relevance of our findings for mature neurons, we investigated the high glucose-induced potentiation of CAP-evoked currents in neurons obtained from adult mice. We took advantage of tgCGRP-eGFP mice, which express enhanced green fluorescence protein (eGFP) in sensory neurons under the control of the CGRP promoter. The latter allowed us to identify CGRP+ peptidergic (nociceptive) neurons in the adult, whose proportion varies from that observed in neonate mice [49]. Similar to our findings from neonate mice, adult CRGP+ DRG neurons maintained in 25 mM glucose had potentiated CAP-evoked currents, quantified as a significant increase in current density and charge (Fig 9). However, in adult neurons, high glucose did not significantly increase the rundown (I_15_/I_max_) of CAP-evoked currents. As expected, adult DRG neurons from WT mice had a larger cell capacitance (*C*_*m*_) than those observed in the neonates (compare Table 1 and Table 3), but we did not observe differences in access resistance and membrane potential between control and high glucose conditions (Table 3).

**Table 3:** Passive membrane properties for cultured DRG neurons from adult wild type mice.

	*V*_*m*_ (mV)	*R*_*in*_ (MΩ)	*C*_*m*_ (pF)	*n*
**CTL**	*-*43.75 ± 0.78	26.13 ± 0.62	24.18 ± 0.57	9
**HG**	-40.35 ± 1.47	24.92 ± 3.73	23.88 ± 2.56	8

Passive membrane properties for cultured DRG neurons from adult wild type mice. Table summarizes the membrane potential (*V*_*m*_), input resistance (*R*_*in*_), and cell capacitance (*C*_*m*_) obtained during whole-cell recording of cultured DRG neurons from adult CGRP- eGFP CD1 mice, maintained in either control or high glucose conditions. Means were compared by unpaired *t*-test, no significant differences were found between the treatments.

## Discussion

In this study, we report RAGE-dependent functional changes that occur in sensory neurons maintained for at least one week in a high glucose condition. We show that 25 mM glucose induces oxidative stress with concomitant changes in electrophysiological responses. The contribution of RAGE and oxidative stress to the pathogenesis of DPN has been particularly considered in the field of diabetes [2,50–52]. However, the cellular and molecular mechanisms involved and their effects on sensory neurons function remain topics of current investigation.

### High glucose potentiated CAP-evoked responses

Previous studies on embryonic cultured DRG neurons exposed to elevated concentration of glucose had increased oxidative stress [24,34], which recapitulates some aspects of the neuron abnormalities observed in animal models of long-standing diabetes. However, these neurons also underwent apoptosis [16,17,24,34]; which contrasts the observations from humans and animal models of diabetes that do not show neuronal loss (sensory or autonomic) at early stages of the disease [53]. This *in vitro* model of sensory neuron damage in diabetes was problematic because it was used with 30 mM glucose as the control condition and 50 mM of glucose as the diabetic condition. These levels of glucose are extremely high to be representative of the physiological blood glucose levels that cells may experience in the periphery. As such, the validity of the findings reported could be questioned. Taking this issue into consideration, we designed a modified version of the cell culture model of sensory neuron used by Vincent et al. [24] to specifically investigate the effect of more relevant levels of glucose (normal and diabetic conditions) on DRG neurons. In our study, we used 5 mM of glucose in control media and 25 mM glucose in high glucose media, concentrations that we have previously reported were sufficient to induce functional changes to autonomic neurons [26,28], and as shown here to DRG neurons. An important distinction from our study versus previous work is that this high glucose concentration does not affect cell viability. It is also important to note that the culture conditions used by us were in complete absence of glial cells (removed by ARA-C treatment) and any diabetes-inducing drugs, such as STZ, which could interfere with normal expression of TRPV1 channels [14]. With these modifications, we demonstrated that high glucose (as short as 1 week), results in cytoplasmic ROS accumulation, and electrophysiological abnormalities (e.g., potentiation of CAP-evoked currents and CAP-evoked depolarizations) in cultured neonatal DRG neurons from mice, as short as 1 week in 25 mM glucose, without inducing apoptotic neuronal loss. In particular, the expression of TRPV1 channels in DRG neurons exposed to 25 mM glucose for up to 10 days was not affected, suggesting that other factors were involved in the regulation of TRPV1 channel function (discussed below).

Since most of our data were obtained from neonate mice, we tested our findings on neurons collected from adult mice as well. Normally, DRG neuron subpopulations undergo maturation at early postnatal life through growth factor-dependent mechanisms, and differentiate into distinct populations of peptidergic and nonpeptidergic neurons. Maturation affects TRPV1 expression, which is down-regulated in nonpeptidergic DRG neurons while peptidergic neurons maintain channel expression, implying that a subpopulation of peptidergic neurons have a greater role in pain processing [49]. Despite developmental differences, this high glucose-mediated effect also occurred in CGRP+ (peptidergic) adult sensory DRG neurons from tgCGRP-eGFP mice. The role of TRPV1 channels in adults is rather complex, and cumulative evidence suggest these channels are good targets for therapeutic interventions. TRPV1-positive DRG fibers innervating the pancreas control islet inflammation and insulin resistance, and a recent report proposes intraperitoneal capsaicin administration as a useful method for the ablation of these fibers and improvement of blood-glucose control in diabetic [54]. Thus, our work on TRPV1 channels in adult DRG neurons under high glucose contributes to better understand TRPV1-mediated abnormalities in DM.

Since TRPV1 channels are highly permeable to Ca^2+^ ions [6], we studied the role of Ca^2+^ on the electrophysiological changes observed in cultured DRG neurons under high glucose. Our Ba-ECF (Ca^2+^-free) experiments in neonatal neurons showed that Ca^2+^ ions drove both, the potentiation and the increased run-down of CAP-evoked currents. We also observed lack of tachyphylaxis, rundown of the currents induced to repetitive agonist application, in Ba-ECF solutions. The latter is not consistent with previous reports in culture DRG neurons, in which CAP-evoked currents underwent even a stronger rundown in Ba-ECF [32]; although, the stronger stimulation (60 s CAP applications) and shorter culture conditions (only 20–30 hr) may be responsible for the contrasting findings. It was previously reported that STZ-induced diabetic rats (early stages) had an approximately three times increase in resting intracellular concentration of Ca^2+^ in DRG neurons from the lumbar segments, which was only reversible in small diameter neurons with help of neurotrophin-3 (NT-3) [55]. Thus, altered Ca^2+^ homeostasis may serve as a marker of early changes in sensory neurons under high glucose. Perhaps the discrepancy that we observed in the run-down of CAP-evoked currents in neonatal versus adult neurons exposed to high glucose reside in altered Ca^2+^ homeostasis. This effect could possibly be based on immature neurons being more sensitive to alterations in Ca^2+^ homeostasis upon activation of TRPV1 channels than neonatal neurons. The deleterious effect of Ca^2+^ entry through TRPV1 channels and its role on neuronal damage was previously considered by Kahya et al. [19]. They showed reduced CAP-evoked Ca^2+^ entry in DRG neurons from STZ-induced diabetic rats that have been treated with melatonin and selenium. These treatments also showed a reduction in oxidative damage-associated markers. Therefore, findings presented here together with previous reports strongly support monitoring Ca^2+^ as an important indicator of sensory abnormalities in early stages of the DM.

### RAGE mediates the potentiation of CAP-evoked currents during high glucose conditions

The up-regulation of RAGE in diabetes has been reported previously in multiple complications associated with the disease, and particularly in DPN including both autonomic [26] and sensory [56,57] neuropathies. Our own research has shown that in the context of diabetic autonomic neuropathy, RAGE expression was increased and required for the accumulation of cytosolic ROS and the inactivation of nicotinic receptors (nAChRs) [26]. Both events were characteristic of the manifestation of symptoms of dysautonomia in diabetic mice [28]. More specific to sensory neuropathy, previous reports showed that mouse models of long-term diabetes had increased RAGE expression in both sensory neurons and Schwann cells [37]. And sural nerve biopsies from patients with diabetic neuropathy had increased RAGE and NF-kB [21]. It has also been demonstrated that the paradoxical loss of pain perception in long-term diabetes required RAGE expression and NF-kB activation [21]. The latter was found to be blunted in RAGE KO mice as well (26). However, a recent report [58] revealed that diabetic RAGE KO mice were protected from developing some symptoms of DPN only at early stages of the disease, but they displayed similar symptoms to wild type mice at long-term diabetes (by 16 weeks). Interestingly, our *in vitro* observations demonstrate that short term exposure (i.e., 1 week) to high glucose was sufficient to potentiate of CAP-evoked currents in DRG neurons, which was prevented in neurons lacking RAGE expression. In our *in vitro* model, RAGE contributes to the early stages of diabetes, however, whether these effects are directly related to sensory changes, or in turn whether these changes are protective or harmful to later stages of diabetes will have to be determined by future studies. Although there is a growing body of evidence supporting the link between RAGE and sensory abnormalities in diabetes, the downstream mechanisms following RAGE activation, and how it affects and triggers sensory neuron malfunction at early stages of the disease remain to be elucidated.

### Intracellular ROS and kinase dependent potentiation of CAP-evoked currents

In the present study, we examined the role of intracellular molecules (e.g. ROS, protein kinases) known to regulate TRPV1 channel function, which are also linked to RAGE signaling. We show that perfusing cells intracellularly with antioxidants prevented the potentiation of CAP-evoked currents. Previous studies have shown that TRPV1 channels are targeted by oxidation, and hydrogen peroxide sensitizes TRPV1 currents in HEK293 cells through the covalent modification of cysteine residues cytoplasmic termini of the channel [59]. Thus, the latter is consistent with the potentiation of CAP-evoked responses in the high glucose condition, which was associated with the accumulation of cytoplasmic ROS. Our cytoplasmic ROS detection revealed that small to large size sensory neurons were affected by glucose-induced oxidative stress, suggesting not only nociceptive (peptidergic) neurons were affected. Therefore, further studies are required to determine whether peptidergic and non-peptidergic neurons are equally affected by the high glucose treatment.

We next concentrated on kinase activity, and particularly explored the involvement of PKCβ and the non-receptor tyrosine kinase Src, which were shown to regulate TRPV1 function. Protein kinases C is required to activate the channel in DRG neurons [60], in addition to potentiating TRPV1 currents by enhancing gating [42,45] Ca^2+^ influx [44]. Notably, the enhanced TRPV1 currents mediated by PKC in co-cultures of DRG and dorsal horn neurons increased sensory input, which resulted in enhanced glutamate release at the DRG central terminal [61]. In the current study, we have concentrated on the PKCβ isotype, which interacts directly with TRPV1 and increases the sensitivity of the channel [42]. Our data suggest that the interaction of TRPV1 with PKCβ may go beyond its physiological regulation and also contributes to the pathology of DPN.

The non-receptor cellular tyrosine kinase c-Src kinase can also regulate TRPV1 channel function. It has been previously reported that phosphorylated (activated) Src kinase co-immunoprecipitated with the TRPV1 channel, demonstrating a physical interaction between these proteins [46]. Functionally, Src regulation of TRPV1 was observed using the Src kinase inhibitor PP2, which blocked CAP-evoked currents in rat colonic DRG neurons and in HEK-293 cells transfected with rat TRPV1 channels [46]. It was reported that application of 10 μM PP2 completely abolished CAP-evoked currents in some colonic DRG neurons, but not in other neurons under same experimental conditions [46]. The latter, is consistent with our own observations in mouse lumbar DRG neurons in which 20 μM, and up to 200 μM (not shown) PP2 did not affect the basal CAP-evoked currents but successfully interfered with the potentiation of the currents in the high glucose condition. Furthermore, it has been reported that phosphorylation of a single tyrosine residue (tyrosine 199) in the TRPV1 channel protein by Src triggers the rapid movement of TRPV1 channels to the surface membrane, potentiating Ca^2+^ influx in response to CAP and sensitizing sensory neurons [47]. However, our data shows that TRPV1 protein expression is unaffected by high levels of glucose despite the potentiation of CAP-evoked currents, suggesting an alternative role of Src in regulating channel function. Collectively, our data strongly suggest that phosphorylation of TRPV1 channels by these kinases may provide potential therapeutic targets to manage sensory abnormalities in diabetic neuropathy.

Both kinases, PKC and Src, are activated downstream from RAGE in various tissues and cell types. In the sensory nervous system, PKC expression and activation is concomitant with RAGE expression [37]. Particularly, in the lumbar DRGs from STZ-induced diabetic rats RAGE-dependent PKC phosphorylation has been linked to pain-related abnormalities. RAGE-dependent PKC phosphorylation of mu-opioid receptors (MOR) leads to receptor desensitization, and consequent loss in opioid antinociceptive efficacy hallmark of diabetic neuropathy [62]. Despite the involvement of RAGE-dependent PKC activation in sensory neurons of diabetic animal models, its role in modulating TRPV1 as a contributor to neuropathy has not been considered so far.

In contrast to PKC, much less is known of Src kinase activation downstream from RAGE. In vascular smooth muscle cells the endogenous RAGE ligand S100B stimulated the Src-dependent tyrosine phosphorylation of caveolin-1 and the activation of the MAPK/NF-κB-STAT3 pathway, resulting in up-regulation of IL-6 and the macrophage-chemoattractant protein 1 (MCP-1) [63]. Consistently, deletion of RAGE expression in pancreatic cancer cells leads to downregulated IL-6 expression, STAT3 localization to mitochondria, as well as the downregulation of the upstream signaling pathways such as phosphorylation of Jak1 and Src [64]. Finally, in vascular cells, evidence suggests that activation of the RAGE-AGE axis during hyperglycemia leads to enhanced activity of the PKC isoforms, particularly β and δ isoforms of PKC (reviewed in [52]).

Taken together, our study provides supportive evidence for the role of RAGE in inducing oxidative stress, abnormal function of TRPV1 channels, and changes in cytosolic signals including Ca^2+^, PCK and Src kinases. We also show that these changes take place at early stages and suggest their possible role in the onset of sensory abnormalities sin diabetes. This study raised new questions to address in future experiments, such as the underlying regulatory mechanisms of cytosolic signals on TRPV1 function, and the importance of these findings on sensory abnormalities at the whole-animal level.
